# RNA-seq-Based Screening in Coal Dust-Treated Cells Identified *PHLDB2* as a Novel Lung Cancer-Related Molecular Marker

**DOI:** 10.1155/2021/1978434

**Published:** 2021-07-19

**Authors:** Deyong Ge, Yuhan Shao, Mengjie Wang, Huihui Tao, Min Mu, Xinrong Tao

**Affiliations:** ^1^Key Laboratory of Industrial Dust Prevention and Control & Occupational Health and Safety, Ministry of Education (Anhui University of Science and Technology), China; ^2^Anhui Province Engineering Laboratory of Occupational Health and Safety, China; ^3^School of Medicine, Anhui University of Science and Technology, No. 168 Taifeng Road, Huainan, Anhui Province, China

## Abstract

Lung cancer is one of the most serious leading cancers with high incidence globally. Identifying molecular markers is key for disease diagnosis and treatment. Coal dust might be important triggering factors in disease development. Here, we first performed RNA-seq-based screening in coal dust treated and nontreated RAW264.7 cell lines. *PHLDB2* was found to be the top differentially expressed gene. By retrieving TCGA lung cancer dataset, we observed that *PHLDB2* showed upregulations in males and smoker patients. Patients with lower *PHLDB2* expression survived longer than those with higher expressions. Furthermore, *PHLDB2* was negatively correlated with EMT makers, and a total of 2.74% mutation rate were observed in 1,059 patients. This finding highlights the critical role of *PHLDB2* in lung cancer development. *PHLDB2* might be a molecular maker for disease diagnosis or treatment.

## 1. Introduction

Lung cancer is one of the most serious leading cancers with high incidence globally. In China, lung cancer ranked the first male and second female cancer incidence according to the recent cancer epidemiology report [1]. During the past twenty years, the crude incidence of lung cancer had been increased dramatically, which caused Chinese cancer bunder the heaviest one around the world. To better control the lung cancer bunder, the government and enterprises made great effort to minimize air pollution, tobacco abuse, emissions of automotive exhausts, and some other restrictions.

Lung cancer is a kind of complex malignant disease with both genetic and strong environment background. Up to date, with the advantage of genome-wide association study and high-throughput next generation sequencing technology, tens of susceptibility loci or gene mutations have been identified in Chinese lung cancer cohorts. Particularly, some disease-driven genes, such as *PIK3CA*, *KRAS*, and *EGFR*, involved in the receptor tyrosine kinase (RTK) pathway, facilitated us to better understand the etiology of disease [2–4]. Even though these genes provide clues in disease development, the accurate molecular mechanism remains largely unknown.

As been widely confirmed, environmental pollution is a high-risk factor for lung cancer. The workers exposed with dust, coal mining, pottery manufacturing, and other kinds of particles, usually containing crystalline silica component, might face the risk of lung cancer [5–7]. The more concentration and longer duration of exposure increased the risk of cancer. Furthermore, when mice delivered with silica particles by inhalation, they developed typical tumor phenotype, suggesting that silica particles might cause lung cancer in rodent animals [8]. The real molecular mechanisms underlying this process are still elusive, but more and more evidence suggest that macrophages and neutrophils are the key cell types that mediate the disease development. The chronic inflammation or fibrosis might occur because of the phagocytic dysfunction [9]. It is largely believed that silica might trigger chronic pulmonary inflammation by inducing chemokine or cytokine like *IL-1*, *IL-6*, and *TNF*, and eventually caused hyperproliferation of epithelial cells [10].

In current study, to better understand how coal dust influence gene expression profile of cultured macrophage cell line, we induce RAW264.7 cells with coal dust then performed RNA-seq, and we identified *PHLDB2* as the top gene that significantly upregulated the coal dust treatment group. To verify our observations, we compared the expression level in the lung cancer and normal control group using the TCGA dataset. Further investigations suggested that those with elevated expression *PHLDB2* might survive shorter than those with lower expression. These findings revealed that *PHLDB2* might contribute to lung cancer by phagocyte dust through macrophage cells.

## 2. Materials and Methods

### 2.1. Cell Culture and Cytotoxicity Test

RAW264.7 cells were cultured in DMEM high sugar medium (Hyclone, USA) with 10% fetal bovine serum (ExCell Bio, China) and 1% penicillin/streptomycin, in humidified incubator at 37°C with 5% CO_2_. The coal dust was collected from a coal mine located in Shanxi Province, China. After grinding, the average diameter of dust particle was lower than 5 *μ*m. Then, coal dust was autoclaved and sonicated and dissolved in DMEM medium with the final concentration 20 mg/mL for long time storage. We cultured RAW264.7 with 0.125 mg/mL, 0.25 mg/mL, 0.5 mg/mL, 1 mg/mL, 2 mg/mL, and measured the cell viability. The CCK8 assay kit (Biosharp, China) was used to evaluate cell viability.

### 2.2. The Process of RNA-seq Data

The total RNA of all 12 cultured cells was extracted with QiagenRNA easy mini kit (Cat No.: 74106, Germany). After controlling the quality and quantity of RNA with nanodrop and electrophoresis, the qualified RNA samples were converted into cDNA, then fragmentation, adaptor ligation, and amplification and were finally loaded into Illumina next generation sequencer.

Skewer was used to remove adaptor sequence and trim those with low quality. Samples with both QC 20% > 95% and QC 30% > 90%, calculated with FastQC, were entered into next step. STAR software was used to align the clean data to the human genome reference, with parameters: --twopassModeBasic|--outSAMstrandFieldintronMotif|--alignSJstitchMismatchNmax 5-1 5 5. Transcripts were assembled, and read counts were generated with StringTie software. Differential gene expressions were detected with edge*R* package (http://bioconductor.org/packages/release/bioc/html/edgeR.html).

### 2.3. Public Data Mining

We downloaded the RNA-seq data of 1,014 non-small-cell lung cancer subjects from Genomic Data Commons (GDC,https://portal.gdc.cancer.gov/). Gene expression data of 578 controls were downloaded from GTEx V8 release version (https://gtexportal.org/home/datasets). For the Kaplan-Meier survival analysis, log ranked survival time was compared between each group. Mean time represents the mean survival time of 50% overall survival probability.

### 2.4. Statistical Analysis

The differential expression was performed with *R* package. Student's *t*-test was used to generate the *p* value in treated and nontreated cell lines. The Wilcoxon rank sum test was used to compare the gene expression difference among cancer and normal groups. Kaplan-Meiersurvival analysis was fixed with “Survival” and “Survminer.” Significance threshold was set at *p* < 0.05.

## 3. Results

### 3.1. Cytotoxicity of Dust-Treated RAW264.7 Cells

**Figure 1:**
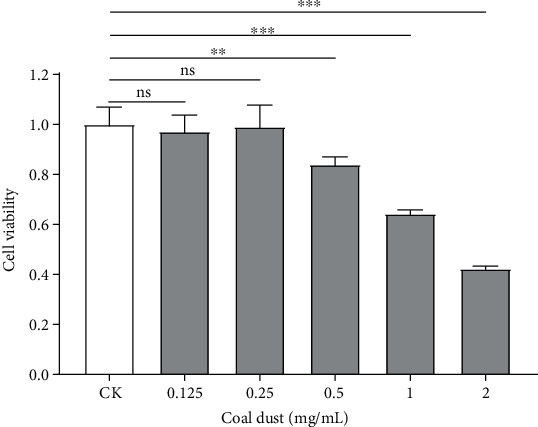
Cell viability was tested in normal RAW264.7 cells and treated with different concentrations of dust-containing medium. All tests were replicated by more than five times. Two tail Student *t*-test *p* value was evaluated the statistical significance (^∗^*p* < 0.05, ^∗∗^*p* < 0.01, ^∗∗∗^*p* < 0.001).

To check whether coal dust affects the viability of RAW264.7 cells, we treated cells with different concentrations of dust-containing medium. CCK-8 assays revealed that low concentration dust showed limited effect on cell viability, while cells were significantly inhibited by coal dust with concentration more than 0.5 mg/mL. At concentration 2.0 mg/mL, the average cell viability decreased 44% when compared with control cells, suggesting coal dust might affect RAW264 in high concentration (Figure 1).

### 3.2. RNA-seq Findings of Dust-Treated and Nontreated Cells

**Figure 2:**
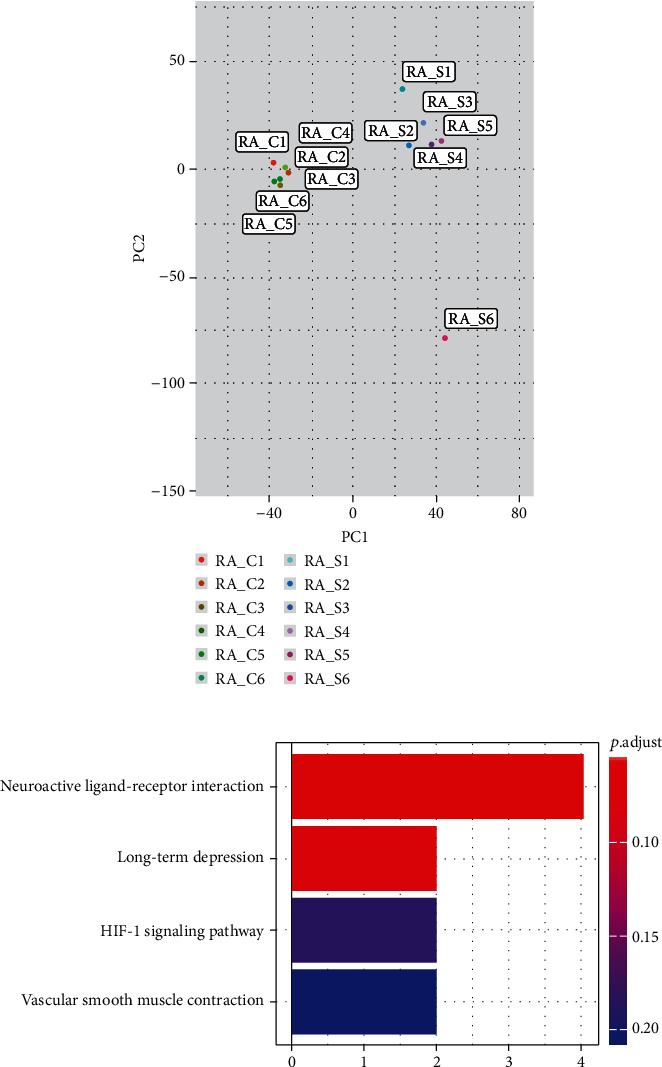
RNA-seq profile of coal dust-treated and nontreated cells. (a) Principle component analysis (PCA) revealed clear separation from treated (RA-S) and nontreated (RA-C) groups. (b) KEGG pathway analysis of 24 differentially expressed genes.

To reveal whether coal dust impact gene expression profile in RAW264.7 cell lines, we performed RNA-seq in six dust-treated and six nontreated cells. We selected coal dust concentration at 1 mg/mL for the transcriptome sequencing, because the cell viability showed strong alteration at this point [11, 12]. We deemed that this concertation was high enough to induce the expression of key mediators that modulated cell viability. The mean clean reads were 47.0 M per sample, with an average mapping rate of 90.1%. PCA analysis showed that the dust-treated group clearly separates from the nontreated group, suggesting reliability of our sequencing data (Figure 2(a)). Using *R* package edge*R*, we calculate expression difference between two groups. With adjusted *p* < 0.05, we found 24 differentially expressed genes (Supplementary Table 1). The most significant gene is *PHLDB2* which upregulated about 28.6 folds. We noticed 91.6% (22/24) gene showed upregulated expression while only two represent decreased expression. For upregulated genes, the second top three were *ATP13A5*, *MC5R*, and *PRKG1*, while *GDA* and *PCDHA2* decreased dramatically.

These genes might modulate the macrophage function in some signaling pathways. We thus carried out a pathway analysis for all differentially expressed genes. Four pathways showed changes with “Neuroactive ligand-receptor interaction” ranked the first one with raw *p* value 1.2 × 10^−3^; however, none passed significance threshold after correction (Figure 2(b)).

These findings suggested that coal dust might induce expression changes in macrophage cell lines with the most differentially expressed genes, *PHLDB2*.

### 3.3. PHLDB2 Might Be Involved in Lung Cancer

**Figure 3:**
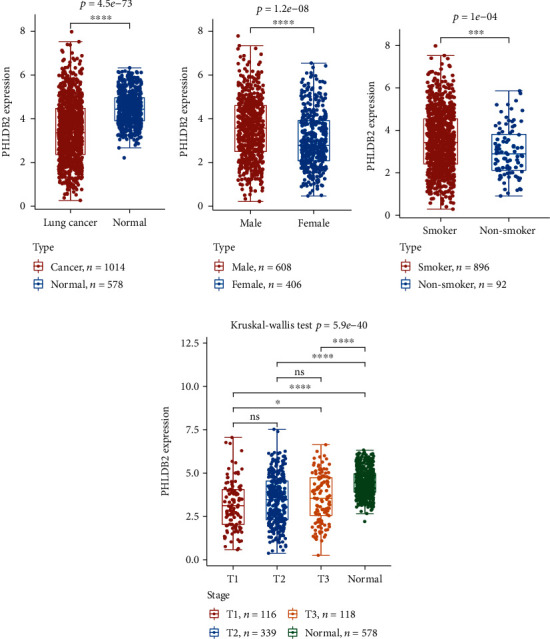
The *PHLDB2* expression in different groups. (a) The *PHLDB2* expression was decreased in cancer patients compared with normal controls. Upregulated *PHLDB2* levels were observed in male (b) and smoker patients (c). (d) The *PHLDB2* expression in different clinical stages. Wilcox ranked test *p* value was used to mark the statistical significance.

*PHLDB2* was the top differentially expressed gene, while it has not been reported with coal dust-exposed lung-related disease. Interestingly, *PHLDB2* was implicated in other kinds of cancer, such asrenal cell carcinoma and gastric and colorectal cancer [13–15]. We then asked whether *PHLDB2* was related with lung cancer. By retrieving TCGA lung cancer expression data and checked expression status in patients, normal controls, smokers, and nonsmokers, the TCGA dataset included 1,014 patients and 578 controls. *PHLDB2* showed significantly lower expression in cancer patients when compared with controls (*p* = 4.5 × 10^−73^, Figure 3(a)). When patients were classified into different clinical stages, we observed an increasing trend that T3 tumors presented with slightly upregulation (Figure 3(d)). Male patients obtained elevated expression than those in female groups (*p* = 1.2 × 10^−8^, Figure 3(b)). Furthermore, *PHLDB2* was significantly higher in smoker cancer patients than in nonsmokers (*p* = 1.0 × 10^−4^, Figure 3(c)).

Interestingly, the expression of *PHLDB2* was decreased in lung cancer tissues, but showed a mosaic change in various kinds of cancer types in a pan-cancer analysis with TCGA data (with upregulation in HNSC (head and neck squamous cell carcinoma), PAAD (pancreatic adenocarcinoma), DLBC (lymphoid neoplasm diffuse large B-cell lymphoma), and some others, while showed downregulation in LUAD (lung adenocarcinoma) and LUSC (lung squamous cell carcinoma) (Supplementary Figure 1). These findings implied that *PHLDB2* might contribute to different cancers with different functions.

**Figure 4:**
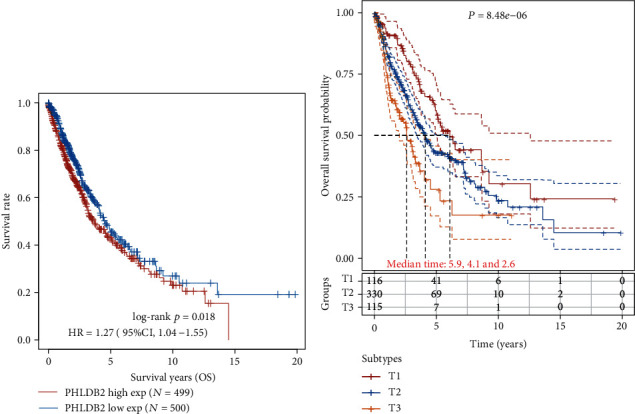
Survival analysis of patients with higher or lower *PHLDB2* expression level (a) and in different clinical stages (b).

The *PHLDB2* expression was associated with survival rate, suggesting higher expression carriers survived shorter than those with lower expression (Figure 4(a)).T3 stage patients expressed slightly higher *PHLDB2* level, and they survived shorter than T1 and T2 stage patients. The median survival time is 5.9 ys, 4.1 ys, and 2.6 ys for T1, T2, and T3 stages, respectively (Figure 4(b)).

**Figure 5:**
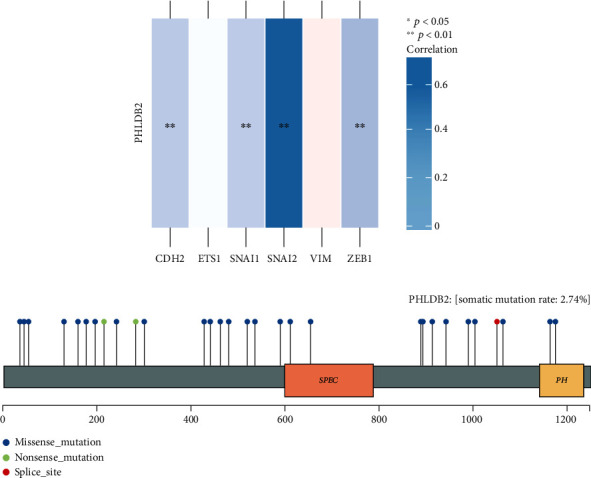
*PHLDB2* expression was negatively correlated with EMT markers (a). *PHLDB2* mutation landscape in lung cancer patients from the TCGA dataset (b).

We noticed that PHLDB2 was negatively correlated with some EMT markers, suggesting that *PHLDB2* might contribute to lung cancer by regulating the EMT process (Figure 5(a)). For the previous key findings in current study, we then asked whether *PHLDB2* mutations contributed to lung cancer. In the TCGA mutation database, we found 29 mutations in the coding and flanking regions of *PHLDB2*, representing 2.74% of 1,059 cancer patients. When looked in detail, the 29 mutations consisted of 25 missense, 3 nonsense, and 1 splicing mutation (Figure 5(b)). Almost all mutations appeared in the patients with relatively lower PHLDB2 expression. These findings from the public dataset implied that *PHLDB2* might be involved in lung cancer development.

## 4. Discussion

In current study, we first executed RNA-seq for coal dust-treated and nontreated macrophage cell line RAW264.7. We found 24 genes that showed differential gene expression between two groups, with *PHLDB2* served as the most significant gene. The 24 genes also showed enrichment trend that was involved in “Neuroactive ligand-receptor interaction” signaling pathway. By retrieving the TCGA public dataset, we observed that *PHLDB2* was significantly decreased in lung cancer patients, while those with higher expression survived longer, suggesting *PHLDB2* might play protective role in caner development. There were 2.74% patients that carried *PHLDB2* mutations. In summary, RNA-seq-based strategies helped us identified *PHLDB2* as a new lung cancer-related maker.

Coal dust is a mixture of copper, lead, zinc, and some other particulate matters 2.5 *μ*m (PM 2.5). Long-term exposure to coal dust might induce severe lung disease, such as pneumoconiosis, pulmonary fibrosis, and even lung cancer. It is commonly believed that lung fibrosis might be mediated by macrophages, neutrophils, and Th17 cells [16], even though the exact molecular mechanism is still under debate. Our study provides some evidence that *PHLDB2* might modulate disease initiation or progression through macrophage, based on RNA-seq-based screening strategy.

*PHLDB2*, also known as pleckstrin homology-like domain family B member 2, has been implicated in the several other types of cancers [17, 18]. The main function of *PHLDB2* is regulating migration by interacting with CLASPS, prickle 1, and liprin *α*1 [19, 20]. This macromolecular complex plays a critical role in assembling focal adhesion and is essential for cell polarization and migration. Indeed, *PHLDB2* has diverse roles in regulating cell migration or EMT (epithelial–mesenchymal transition). We found that *PHLDB2* was negatively correlated with several EMT markers, suggesting that *PHLDB2* might be involved in the EMT process. However, this point cannot be figured out in current study because of our limited data. A recent report revealed that NOTCH3 might promote cell proliferation, monolayer formation, or cell invasion of gastric carcinogenesis through *PHLDB2*. Furthermore, *PHLDB2* signals can be transduced into Akt signaling pathway, because *PHLDB2* knockdown inhibited the activation of Akt and mTOR [14]. Meanwhile, pan-cancer analysis suggested *PHLDB2* upregulated or down regulated in different types of cancer, implying that *PHLDB2* might contribute to different cancers with different functions. There were also some other possibilities that the *PHLDB2* expression alteration was just the consequence of some signaling pathways, like the miR-875-5p-NOTCH3- pathway, which might be regulated by several undetermined factors. Even *PHLDB2* might be holding the same function in EMT, while the network of controlling EMT is really far beyond the scope of our current study and also the other types of cancer. Particularly, recent bioinformatic strategies revealed interesting findings that some immune-related genes are linked to lung adenocarcinoma. Whether *PHLDB2* is connected to these genes or signaling pathways needs further investigation.

The limitation of this study mainly stands in two points. First, *PHLDB2* was found in the RAW264.7 macrophage cell line. How *PHLDB2* contributes to lung cancer? Does it the signal that macrophage communicates with epithelial cell? Even the typical phenotype of coal dust inhalation was lung fibrosis, what is the functions of *PHLDB2* in fibrosis? Second, most of our findings were based on statistical analysis of public dataset, and further investigation was fundamental to confirm these results.

In summary, using RNA-seq-based screening strategy, we detected that *PHLDB2* might be a potential lung cancer-related marker. These findings will help us to better understand the etiology of coal dust-induced lung disease.

## Data Availability

Public data can be accessed from https://portal.gdc.cancer.gov.

## Supplementary Materials

Supplementary 1 Supplementary Table 1: Twenty-four differentially expressed genes.

Supplementary 2 Supplementary Figure 1: The *PHLDB2* expression in various kinds of cancers from TCGA and TCGA + GTEx datasets. Log(TPM+1) expression levels were compared between cases and control groups. (^∗^*p* < 0.05, ^∗∗^*p* < 0.01, ^∗∗∗^*p* < 0.001). ACC: adrenocortical carcinoma; BLCA: bladder urothelial carcinoma; BRCA: breast invasive carcinoma; CESC: cervical squamous cell carcinoma and endocervical adenocarcinoma; CHOL: cholangiocarcinoma; COAD: colon adenocarcinoma; DLBC: lymphoid neoplasm diffuse large B-cell lymphoma; ESCA: esophageal carcinoma; GBM: glioblastoma multiforme; HNSC: head and neck squamous cell carcinoma; KICH: kidney chromophobe; KIRC: kidney renal clear cell carcinoma; KIRP: kidney renal papillary cell carcinoma; LAML: acute myeloid leukemia; LGG: brain lower grade glioma; LIHC: liver hepatocellular carcinoma; LUAD: lung adenocarcinoma; LUSC: lung squamous cell carcinoma; MESO: mesothelioma; OV: ovarian serous cystadenocarcinoma; PAAD: pancreatic adenocarcinoma; PCPG: pheochromocytoma and paraganglioma; PRAD: prostate adenocarcinoma; READ: rectum adenocarcinoma; SARC: sarcoma; SKCM: skin cutaneous melanoma; STAD: stomach adenocarcinoma; TGCT: testicular germ cell tumors; THCA: thyroid carcinoma; THYM: Thymoma; UCEC: uterine corpus endometrial carcinoma; UCS: uterine carcinosarcoma; UVM: uveal melanoma.
