# Supplementary material for: RNA-seq-Based Screening in Coal Dust-Treated Cells Identified PHLDB2 as a Novel Lung Cancer-Related Molecular Marker
Source: Biomed Res Int. 2021 Jul 19;2021:1978434. doi: 10.1155/2021/1978434 (PMC8314042; doi:10.1155/2021/1978434)
Supplement: Supplementary 1 — Supplementary Table 1: Twenty-four differentially expressed genes. [file 1978434.f1.docx]

| Supplymentary table 1.Twenty-four differentially expressed genes | | | | |
| --- | --- | --- | --- | --- |
| Symbol | logFC | logCPM | PValue | FDR |
| PHLDB2 | 4.84 | 4.63 | 9.32E-08 | 1.43E-03 |
| ATP13A5 | 4.8 | 4.61 | 1.44E-07 | 1.43E-03 |
| MC5R | 4.6 | 4.52 | 9.06E-07 | 3.88E-03 |
| PRKG1 | 4.62 | 4.53 | 7.78E-07 | 3.88E-03 |
| SSTR5 | 4.32 | 4.4 | 9.73E-06 | 3.24E-02 |
| CHRND | 4.15 | 4.33 | 3.14E-05 | 3.61E-02 |
| CLDN4 | 4.16 | 4.34 | 2.90E-05 | 3.61E-02 |
| CYP2B10 | 4.12 | 4.32 | 3.84E-05 | 3.61E-02 |
| EBF3 | 4.14 | 4.33 | 3.38E-05 | 3.61E-02 |
| EPO | 4.21 | 4.36 | 2.05E-05 | 3.61E-02 |
| FBXO41 | 4.24 | 4.37 | 1.69E-05 | 3.61E-02 |
| GDA | -2.81 | 4.73 | 2.23E-05 | 3.61E-02 |
| IGF1R | 2.93 | 4.66 | 3.85E-05 | 3.61E-02 |
| KRT86 | 4.2 | 4.35 | 2.22E-05 | 3.61E-02 |
| MYOZ3 | 4.24 | 4.37 | 1.71E-05 | 3.61E-02 |
| NPPB | 2.56 | 4.87 | 2.20E-05 | 3.61E-02 |
| PTGDS | 4.17 | 4.34 | 2.76E-05 | 3.61E-02 |
| TBR1 | 2.55 | 4.92 | 1.32E-05 | 3.61E-02 |
| TMEFF2 | 4.15 | 4.33 | 3.20E-05 | 3.61E-02 |
| TRPV1 | 4.11 | 4.32 | 3.99E-05 | 3.62E-02 |
| TSPAN7 | 4.09 | 4.31 | 4.65E-05 | 3.98E-02 |
| PSG29 | 4.07 | 4.31 | 5.19E-05 | 4.09E-02 |
| SCGB3A1 | 2.8 | 4.68 | 5.02E-05 | 4.09E-02 |
| PCDHA2 | -4.04 | 4.28 | 5.96E-05 | 4.58E-02 |
| Symbol: Gene name; FC: Fold Change; CPM:Counts Per Million; FDR: False Discovery Rate | | | | |
