# Supplementary material for: RNA-seq-Based Screening in Coal Dust-Treated Cells Identified PHLDB2 as a Novel Lung Cancer-Related Molecular Marker
Source: Biomed Res Int. 2021 Jul 19;2021:1978434. doi: 10.1155/2021/1978434 (PMC8314042; doi:10.1155/2021/1978434)
Supplement: Supplementary 2 — Supplementary Figure 1: The PHLDB2 expression in various kinds of cancers from TCGA and TCGA + GTEx datasets. Log(TPM+1) expression levels were compared between cases and control groups. (∗p < 0.05, ∗∗p < 0.01, ∗∗∗p < 0.001). ACC: adrenocortical carcinoma; BLCA: bladder urothelial carcinoma; BRCA: breast invasive carcinoma; CESC: cervical squamous cell carcinoma and endocervical adenocarcinoma; CHOL: cholangiocarcinoma; COAD: colon adenocarcinoma; DLBC: lymphoid neoplasm diffuse large B-cell lymphoma; ESCA: esophageal carcinoma; GBM: glioblastoma multiforme; HNSC: head and neck squamous cell carcinoma; KICH: kidney chromophobe; KIRC: kidney renal clear cell carcinoma; KIRP: kidney renal papillary cell carcinoma; LAML: acute myeloid leukemia; LGG: brain lower grade glioma; LIHC: liver hepatocellular carcinoma; LUAD: lung adenocarcinoma; LUSC: lung squamous cell carcinoma; MESO: mesothelioma; OV: ovarian serous cystadenocarcinoma; PAAD: pancreatic adenocarcinoma; PCPG: pheochromocytoma and paraganglioma; PRAD: prostate adenocarcinoma; READ: rectum adenocarcinoma; SARC: sarcoma; SKCM: skin cutaneous melanoma; STAD: stomach adenocarcinoma; TGCT: testicular germ cell tumors; THCA: thyroid carcinoma; THYM: Thymoma; UCEC: uterine corpus endometrial carcinoma; UCS: uterine carcinosarcoma; UVM: uveal melanoma. [file 1978434.f2.pdf]

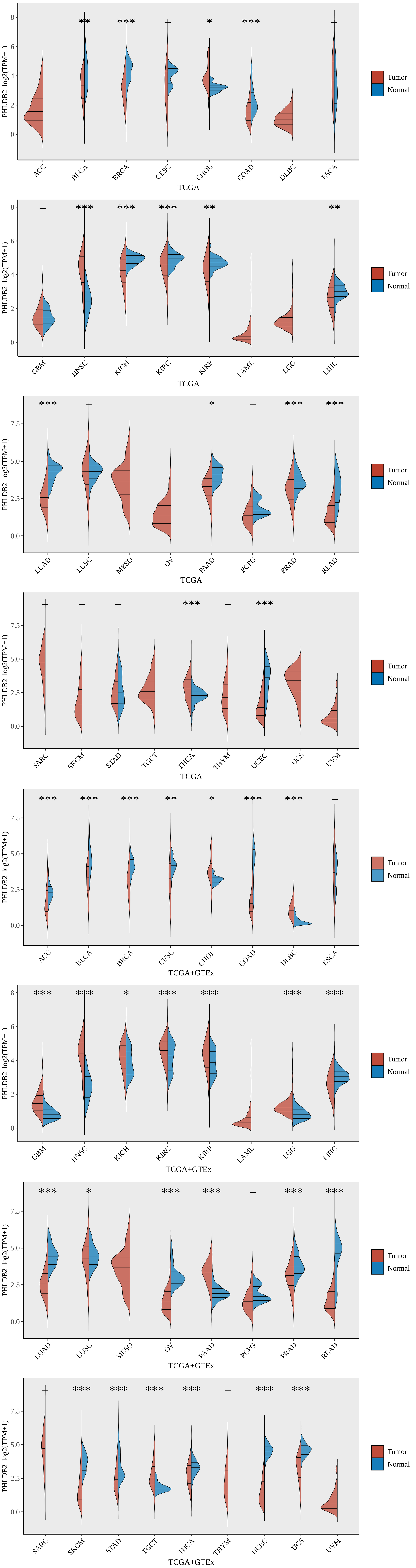
PHLDB2 log2(TPM+1)
Tumor
Normal
ACC BLCA BRCA CESC CHOL COAD DLBC ESCA
TCGA
GBM HNSC KICH KIRC KIRP LAML LGG LIHC
TCGA
LUAD LUSC MESO OV PAAD PCPG PRAD READ
TCGA
SARC SKCM STAD TGCT THCA THYM UCEC UCS UVM
TCGA
ACC BLCA BRCA CESC CHOL COAD DLBC ESCA
TCGA+GTEx
GBM HNSC KICH KIRC KIRP LAML LGG LIHC
TCGA+GTEx
LUAD LUSC MESO OV PAAD PCPG PRAD READ
TCGA+GTEx
SARC SKCM STAD TGCT THCA THYM UCEC UCS UVM
TCGA+GTEx
